# Intracranial Pressure Is a Determinant of Sympathetic Activity

**DOI:** 10.3389/fphys.2018.00011

**Published:** 2018-02-08

**Authors:** Eric A. Schmidt, Fabien Despas, Anne Pavy-Le Traon, Zofia Czosnyka, John D. Pickard, Kamal Rahmouni, Atul Pathak, Jean M. Senard

**Affiliations:** ^1^Institut des Maladies Métaboliques et Cardiovasculaires, I2MC, Institut National de la Santé et de la Recherche Médicale, Université de Toulouse, Toulouse, France; ^2^Department of Neurosurgery, University Hospital of Toulouse, Toulouse, France; ^3^Department of Clinical Pharmacology, University Hospital of Toulouse, Toulouse, France; ^4^Department of Neurology, University Hospital of Toulouse, Toulouse, France; ^5^Brain Physics Lab, Academic Neurosurgery, University of Cambridge, Cambridge, United Kingdom; ^6^Departments of Pharmacology, University of Iowa, Iowa City, IA, United States

**Keywords:** intracranial pressure, autonomic nervous system, baroreflex, microneurography, translational medical research, physiology

## Abstract

Intracranial pressure (ICP) is the pressure within the *cranium*. ICP rise compresses brain vessels and reduces cerebral blood delivery. Massive ICP rise leads to cerebral ischemia, but it is also known to produce hypertension, bradycardia and respiratory irregularities due to a sympatho-adrenal mechanism termed Cushing response. One still unresolved question is whether the Cushing response is a non-synaptic acute brainstem ischemic mechanism or part of a larger physiological reflex for arterial blood pressure control and homeostasis regulation. We hypothesize that changes in ICP modulates sympathetic activity. Thus, modest ICP increase and decrease were achieved in mice and patients with respectively intra-ventricular and lumbar fluid infusion. Sympathetic activity was gauged directly by microneurography, recording renal sympathetic nerve activity in mice and muscle sympathetic nerve activity in patients, and gauged indirectly in both species by heart-rate variability analysis. In mice (*n* = 15), renal sympathetic activity increased from 29.9 ± 4.0 bursts.s^−1^ (baseline ICP 6.6 ± 0.7 mmHg) to 45.7 ± 6.4 bursts.s^−1^ (plateau ICP 38.6 ± 1.0 mmHg) and decreased to 34.8 ± 5.6 bursts.s^−1^ (post-infusion ICP 9.1 ± 0.8 mmHg). In patients (*n* = 10), muscle sympathetic activity increased from 51.2 ± 2.5 bursts.min^−1^ (baseline ICP 8.3 ± 1.0 mmHg) to 66.7 ± 2.9 bursts.min^−1^ (plateau ICP 25 ± 0.3 mmHg) and decreased to 58.8 ± 2.6 bursts.min^−1^ (post-infusion ICP 14.8 ± 0.9 mmHg). In patients 7 mmHg ICP rise significantly increases sympathetic activity by 17%. Heart-rate variability analysis demonstrated a significant vagal withdrawal during the ICP rise, in accordance with the microneurography findings. Mice and human results are alike. We demonstrate in animal and human that ICP is a reversible determinant of efferent sympathetic outflow, even at relatively low ICP levels. ICP is a biophysical stress related to the forces within the brain. But ICP has also to be considered as a physiological stressor, driving sympathetic activity. The results suggest a novel physiological ICP-mediated sympathetic modulation circuit and the existence of a possible intracranial (i.e., central) baroreflex. Modest ICP rise might participate to the pathophysiology of cardio-metabolic homeostasis imbalance with sympathetic over-activity, and to the pathogenesis of sympathetically-driven diseases.

## Introduction

Intracranial pressure (ICP) is a complex brain modality that determines cerebral perfusion pressure (CPP), which is the difference between arterial blood pressure (ABP), and ICP. Raised ICP reduces CPP and blood delivery to the brain that jeopardizes cerebral function and organismal survival in many species. A massive rise in ICP is also known to produce an increase in ABP, bradycardia and respiratory irregularities termed Cushing response (Cushing, 1901). This mechanism is generally considered to be an agonal and terminal event occurring in extreme condition of brainstem ischaemia leading to a sympatho-adrenal response (McGillicuddy et al., 1978; Nagao et al., 1984; Van Loon et al., 1993). However, it is still debated whether the Cushing response is an acute pathological response to brain ischemia or part of an important physiological reflex mechanism for ABP regulation (Paton et al., 2009). This assumption is based on the following findings: (i) experimental studies show that ABP is sensitive to small changes in ICP in dogs (Jeffers et al., 1937; Dickinson and McCubbin, 1963), cats (Nakamura et al., 1987), and rabbits (Edvinsson et al., 1971; Donnelly et al., 2017), (ii) demonstration in non-human primates of a short lasting, repeatable and non-terminal vasomotor response to modest ICP increase (Fitch et al., 1977), (iii) presence of pressure receptive area located in the lower brainstem (Thompson and Malina, 1959; Hoff and Reis, 1970; Doba and Reis, 1972; Reis and Doba, 1972), (iv) bedside evidence that modest and controlled ICP rise in non-lethal conditions might yield a concomitant and reversible rise in ABP (Dickinson, 1981), (v) modest and gradual ICP rise in awake patients produces an increase in ABP and heart rate (HR) variance (Schmidt et al., 2005), (vi) demonstration of an interdependency between ICP and HR variability suggesting a centrally organized neural control (Hu et al., 2007), (vii) ICP rise augments ICP variability and reduces ICP complexity (Santamarta et al., 2012), and finally (viii) the suggested ability of the central nervous system to modulate the set point of the arterial baroreceptor reflex (Osborn, 2005). Hence, animal and clinical studies suggest that ICP change may modulate systemic hemodynamics probably *via* the sympathetic nervous system (SNS). But direct demonstration that ICP modulates SNS is still lacking.

In the present study, we hypothesize that modest ICP changes drive sympathetic activity. Using microneurography, state-of-the-art technique to measure *in vivo* direct and dynamic post-ganglionic SNS activity, we performed two different sets of experiments in mice and patients. During controlled modest ICP increase and decrease, sympathetic activity was measured directly by renal sympathetic nerve activity (RSNA) in mice and by muscle sympathetic nerve activity (MSNA) in patients. SNS activity was also indirectly gauged by HR variability analysis in animal and human.

## Methods

### Animal experiments

All studies were approved by the University of Iowa Animal Research Committee. Experiments were performed in adult male mice (C57BL/6J; Jackson Laboratory, USA). Experiments were performed by trained and authorized staff (KR, FD). Mice were anesthetized and placed in a stereotactic device (Kopf Instruments). A sterile cerebro-ventricular cannula was implanted into the right lateral ventricle and sealed on the skull with dental cement. This cannulation was used to infuse fluid in the ventricle to increase ICP. Mice were given 5–7 days to recover from ventricular cannulation before the experimental procedure.

### ICP increase in animal

Mice were anesthetized by i.p. injection of ketamine (91 mg.kg^−1^) plus xylazine (9.1 mg.kg^−1^) and intubated (PE-50) to allow spontaneous respiration of oxygen-enriched room air. Body temperature was kept constant at 37.5°C using a surgical heat lamp and a heat pad. The right jugular vein was cannulated to maintain anesthesia with α-chloralose (initial dose 25 mg.kg^−1^, sustaining dose of 6 mg.kg^−1^.h^−1^). This standard anesthetic protocol has little impact on SNS activity and was shown to be adapted to RNSA recording (Rahmouni et al., 2005). The skull of the mouse was perforated in the left frontal region and a pressure sensor (CODMAN, USA) was inserted 0.5 cm deep within the brain and sealed with dental cement. The cerebro-ventricular cannula were connected to a 1 ml syringe with a micro-renathane tubing (Braintree Scientific, Braintree, MA, USA) filled with artificial cerebrospinal fluid (CSF). Temperature of the artificial CSF was controlled by immersion of the tubing in a water container at 37.5°C. The artificial CSF was injected with a controlled micro-debit pump at a rate from 5 to 50 μl.min^−1^ (Syringe pumps for mass spectrometry, Harvard Apparatus, Holliston, Massachusetts, USA). ICP was recorded at baseline for 10 min; then the ventricle was infused with artificial CSF at body temperature with a constant rate.

### Arterial blood pressure, respiratory, and sympathetic recording in animal

Physiological parameters were continuously monitored. ABP and HR were measured from a femoral arterial line (AD Instruments, Castle Hill, Australia). Respiratory movements were measured with a piezoelectric sensor placed on rib cage (AD Instruments, Castle Hill, New South Wales, Australia).

After a left lumbar dissection, the kidney was exposed *via* a retroperitoneal approach. A 36-gauge platinum–iridium bipolar electrode (Cooner Wire Co, Chadsworth, California, USA) placed on the nerves of the left kidney in order to record RSNA. The procedure is detailed by Rahmouni et al. (2005). Once optimal recording parameters were established, the nerve fibers were fixed to the electrodes with silicone gel (Kwik-Sil, World Precision Instruments Inc, Sarasota, Florida, USA). The nerve electrodes were attached to a high-impedance probe (HIP-511, Grass Instruments Co, Quincy, Massachusetts, USA). The signal was amplified 10^5^ times with a Grass P5 AC preamplifier and filtered at both low (100 Hz) and high-frequency (1,000 Hz) cut-off. The signal was also routed to a Powerlab analog–digital converter (Ad Instruments, Castle Hill, Australia) for recording and off-line data analysis on a computer. The signal was amplified, filtered, rectified, integrated and RSNA was expressed in bursts.s^−1^ (Rahmouni et al., 2005). At the end of the study, mice were euthanized with a lethal dose of ketamine and xylazine. The integrated voltage after death (background noise) was subtracted from the total integrated voltage to calculate real RSNA.

**Figure 1:**
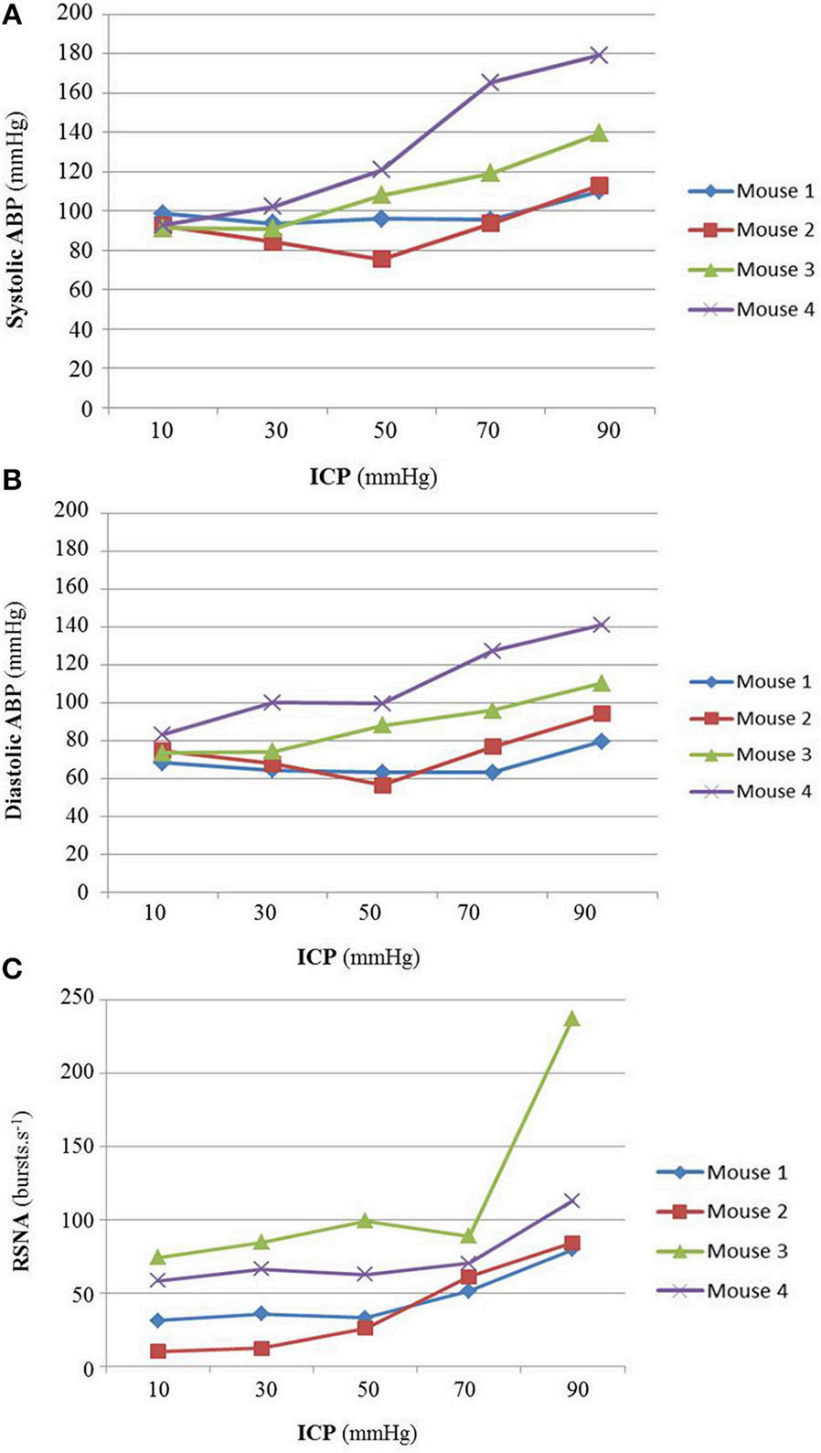
**(A)** Animal results for massive ICP increase (*n* = 4). Individual animal results of systolic ABP **(A)**, diastolic ABP **(B)**, and RSNA **(C)** during massive ICP increase. The Cushing response was elicited at ICP 50 mmHg, with an increase in ABP (cf. **A,B**) and a steep augmentation of RSNA due to massive SNS activation (cf. **C**) leading to respiratory arrest and to a quick death. For our proper study, the upper ICP threshold was set at 40 mmHg, before the onset of the Cushing response. No statistical analysis has been performed.

In a preliminary study (*n* = 4), a massive ICP rise was performed. ICP was slowly increased to reach diastolic ABP in order to validate that our animal model elicits a Cushing response and to define the upper ICP threshold that do not produce any significant ABP increase (*cf*. Figures 1A–C). ICP over 50 mmHg yields a Cushing response with systolic and diastolic ABP increase (*cf*. Figures 1A,B), steep augmentation of RSNA due to massive SNS activation (*cf*. Figure 1C) and respiratory arrest leading to a quick death. Therefore, the upper ICP threshold for our mice study was set at 40 mmHg.

For the animal experiments (*n* = 15) a 10-min baseline period was recorded, then ICP was slowly increased up to 40 mmHg. Once this upper ICP threshold has been reached, the artificial CSF infusion was stopped and ICP slowly decreased to a steady post-infusion pressure.

Off-line, dynamic changes of RSNA were measured during 60 s epochs at baseline ICP and at various ICP levels: 10, 20, 30, and 40 mmHg. RSNA was also measured after the end of the infusion when ICP reached a steady post-infusion level.

### Heart rate variability analysis in animal

The ABP signal was digitized at 1,000 Hz and processed (Labchart 5.0, AD Instruments, Castle Hill, Australia). Pulse interval (PI, surrogate for R-R interval) was calculated between two successive dABP/dt max points. Each recording was visualized to select one period without erratic fluctuations of enough duration (>51.2 s) for each session. The rhythmicity of PI was evaluated by power spectral analysis using a fast Fourier transform (FFT) (HR variability analysis, AD Instruments, Castle Hill, New South Wales, Australia). Briefly, the evenly spaced sampling allowed for direct spectral analysis using a FFT algorithm of a 1,024 point series, corresponding to a 51.2 s period, using a sampling rate of 20 Hz. Two bands of FFT the spectrum were considered. The first zone covers the 0.15–0.60 Hz range of the PI spectrum and corresponds to the LF zone. The second zone covers the 2.5–5.0 Hz range of the PI spectrum and corresponds to the HF zone. HF is a reasonable index of parasympathetic activity whereas LF and LF/HF are supposed to be related to sympathetic activity (Task Force of the European Society of Cardiology and the North American Society of Pacing and Electrophysiology, 1996). In our study, HR variability results were safely interpreted as an indication of vagal withdrawal (HF) (Marchi et al., 2016a). One mouse was excluded from the analysis due to heart rate disorders.

### Human experiments

The study was approved by the competent authority and registered by ClinicalTrials.gov under n° NCT01776801. Ten patients suspected of normal pressure hydrocephalus (sex ratio 7/3, mean age 65.2 ± 4.6 years, mean body mass index 25.6 ± 1.2 kg.m^−2^) were included in this prospective study after they gave their informed and written consent. The experiment was performed in the autonomic unit at Toulouse University Hospital by trained staff (AP, FD).

### ICP increase in human

Lumbar infusion studies are procedures performed in awake patients to assess CSF hydrodynamics. This procedure is painless (Manet et al., 2016) and recommended by experts (Marmarou et al., 2005) as part of the clinical management of patients with possible normal pressure hydrocephalus. The patient was lying horizontally, in left lateral recumbent. A lumbar puncture (L4-L5 or L5-S1) was performed with a 19G needle. Then the needle was connected, *via* a 3 way tap and a stiff tube filled with normal saline, to an infusion pump and a pressure transducer. CSF pressure, once zeroed at the level of the third ventricle, is a surrogate marker of ICP (Lenfeldt et al., 2007). CSF pressure was digitized and stored in a computer for off-line analysis. ICP was measured at baseline for 10 min. Then the lumbar subarachnoid space was slowly infused with normal saline solution (0.9%) at room temperature (20°C) at a rate of 1.5 ml.min^−1^. Subsequently ICP increased until a steady-state plateau has been reached. This plateau ICP represents the equilibrium at which the mock CSF infusion flow is reabsorbed through the arachnoid granulations into the venous blood stream. For safety reasons, ICP was always kept below 40 mmHg. Once the plateau ICP has been achieved, the saline infusion was stopped and ICP slowly decreased to a steady post-infusion pressure.

### Arterial blood pressure, respiratory, and sympathetic recording in human

Physiological parameters were continuously monitored. HR was measured by EKG (AD Instruments, Castle Hill, Australia), ABP using photoplethysmography (Finapres Medical System BV, Amsterdam, The Netherlands), oxygen saturation with a pulse oxymeter (AD Instruments, Castle Hill, New South Wales, Australia) and respiratory movement with a thoracic belt (Pneumotrace II, UFI, California, USA).

Muscle sympathetic nerve activity (MSNA) was measured in patients during the infusion test under carefully standardized conditions (White et al., 2015). Signal was recorded by a sterile single-use tungsten microelectrode (shaft diameter, 200 μm) inserted selectively in sympathetic efferent fibers around the right fibular nerve. A subcutaneous reference electrode was inserted 2–3 cm away from the recording microelectrode. Electrode was manipulated until a satisfactory signal was recorded. Once positioned, the recording microelectrode allows the collection of the electrical activity of sympathetic contingent, which appears as a sequence of electrical bursts. In the absence of sensory stimuli and muscle movement, the potential difference measured between the 2 microelectrodes is the sum of the electrical activity of sympathetic fibers of the peripheral muscle vessels. To validate the correct positioning of the microelectrode recording, two tests were carried out: cutaneous stimulation and apnoea. Cutaneous stimulation of the fibular nerve distribution territory should not cause changes in the neurogram activity. Voluntary apnoea and expiration was performed by the subject to activate SNS and should produce an increase in the frequency and amplitude of measured bursts. These tests were done before and after the infusion to verify the correct positioning of the microelectrode. The neural signal was amplified, filtered, rectified and integrated to obtain a neurogram identifying trains of SNS discharge visualized as a sequence of bursts. MSNA was expressed both in bursts.min^−1^ and bursts/100 heart beats (HB).

Off-line, dynamic changes of sympathetic nerve activity were measured during 60 s epochs at baseline ICP and at various ICP levels: 10, 15, 20, and 25 mmHg. MSNA activity was also measured 5 min after the end of infusion when ICP reached a steady post-infusion level.

### Heart rate variability analysis in human

RR intervals derived from EKG were digitized. Series of 512 equidistant values were sampled at 2 Hz, generated and stored in a computer for off-line analysis. Spectral analysis was performed using a FFT algorithm (HR variability analysis, AD Instruments, Castle Hill, Australia) at baseline and during the ICP plateau. Each recording was examined to verify stationarity of the signal using autoregression coefficient monitoring and elimination of artifacts. In accordance with current guidelines for HR variability analysis, the integration of the spectral modulus values from 0.004 to 1 Hz was used to estimate the total power (TP). The integration of the spectral energy contained in consecutive bands between 0.04 and 0.15 Hz from HR spectrum was used to measure low-frequency (LF) band. The integration of spectral energy contained between 0.15 and 0.40 Hz from HR spectrum has been used to measure high frequency (HF) band. With the limits mentioned in the mice section, the relative energy in HF band (nu) of HR spectrum [(HF spectral modulus/TP spectral modulus) ^*^ 100] was used as an index of parasympathetic activity. LF expressed in normalized units [(LF spectral modulus/TS spectral modulus)^*^100] and the ratio between LF/HF are supposed to be related to sympathetic activity (Task Force of the European Society of Cardiology and the North American Society of Pacing and Electrophysiology, 1996). In our study, HR variability results were safely interpreted as an indication of vagal withdrawal (HF) (Marchi et al., 2016a).

### Statistics

This study was designed to test the effect of ICP on SNS activity in mice and human. Analyses of animal and clinical data were carried out off line by two different investigators and values were averaged for each period. The first analysis was performed by one of the investigator (FD); the second analysis was done by a research assistant. Both investigators were blind from the ICP changes.

Data are expressed as mean ± standard error of the mean (sem). Equality of variance was performed with Bartlett's test. Parametric analyses were performed with repeated measures ANOVA with Tukey's post-test, and non-parametric analyses were performed with a Friedman test with Dunns' post-test. All testing was two-sided and a *p* < 0.05 was considered to indicate statistical significance. All analyses were completed with Graphpad Prism, version 5.04 for PC.

## Results

### Effect of modest ICP increase on sympathetic activity in animal

**Figure 2:**
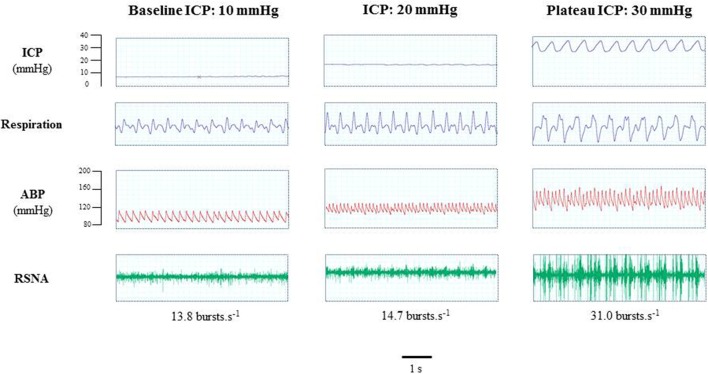
Example of a mouse recording with raw signals of ICP, respiration, ABP and sympathetic activity during modest ICP rise. Example of a mouse raw signal of ICP, respiration, ABP and neurogram of RSNA at baseline ICP (≈ 10 mmHg), ICP (≈ 20 mmHg) and plateau ICP (≈ 30 mmHg).

**Figure 3:**
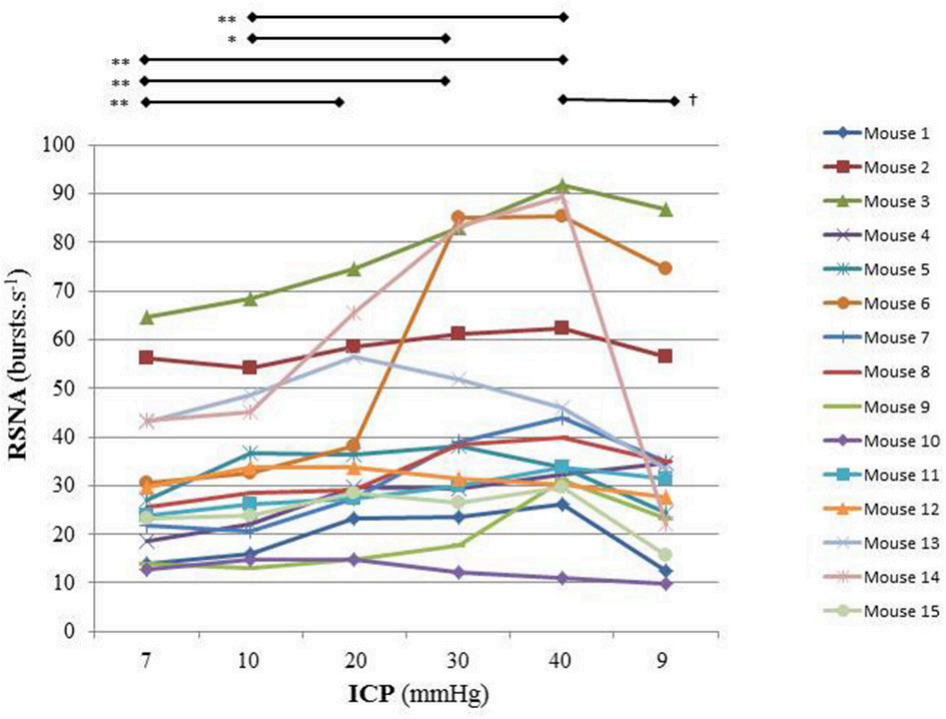
Individual sympathetic activity in each mouse during modest ICP increase and decrease (*n* = 15). Statistical analysis was performed using ANOVA for repeated measures with Tukey's post-test. ^*^p < 0.05, ^**^p < 0.01 vs. baseline and ^†^*p* < 0.05 vs. plateau.

**Figure 4:**
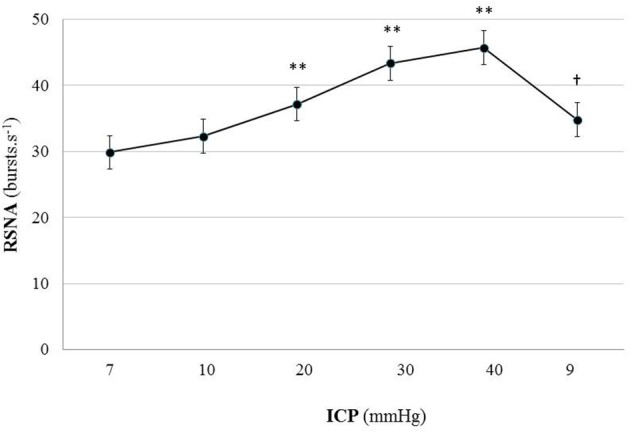
Graph of RSNA changes during modest ICP increase and decrease in animal (*n* = 15). Mean ± sem values of RSNA values at various ICP levels. Statistical analysis was performed using ANOVA for repeated measures with Tukey's post-test. ^**^p < 0.01 vs. baseline and ^†^*p* < 0.05 vs. plateau.

Figure 2 is an example of a mouse recording with raw signals of ICP, respiration, ABP and RNSA. Figure 3 displays individual RSNA results in each mouse during modest ICP increase and decrease. Figure 4 is a graph of RSNA vs. ICP. Table 1 shows animal results (RSNA, systolic and diastolic ABP, HR, respiratory rate and amplitude) during modest ICP increase and decrease. Table 2 depicts the results of spectral analysis of HR variability at baseline and plateau ICP in mice. Statistical significance is indicated in Figures 3, 4 and Tables 1, 2.

**Table 1:** Results of sympathetic activity and systemic parameters during modest ICP increase and decrease in animal (*n* = 15).

**ICP**	**Baseline**	**10 mmHg**	**20 mmHg**	**30 mmHg**	**Plateau**	**Post infusion**
ICP (mmHg)	6.65 ± 0.67	11.72 ± 0.46	21.51 ± 0.43^**^	30.96 ± 0.23^**^	38.57 ± 1.02^**^	9.14 ± 0.83^††^
RSNA (bursts.s^−1^)	29.87 ± 4.00	32.29 ± 4.10	37.17 ± 4.69^**^	43.36 ± 6.25^**^	45.73 ± 6.42^**^	34.82 ± 5.65^†^
ABPs (mmHg)	94.21 ± 7.25	90.22 ± 7.35	92.06 ± 6.26	94.90 ± 6.24	97.52 ± 6.34	90.35 ± 6.54
ABPd (mmHg)	68.67 ± 6.19	65.72 ± 6.35	67.06 ± 5.39	69.17 ± 4.98	71.36 ± 4.76	67.84 ± 5.41
HR (beats.min^−1^)	307.14 ± 17.59	311.51 ± 18.20	308.76 ± 17.00	321.47 ± 17.64	329.74 ± 16.43	356.57 ± 17.80^**^
Resp. rate (min^−1^)	113.94 ± 5.21	111.97 ± 7.44	114.66 ± 5.67	123.79 ± 5.89	122.52 ± 5.16	119.65 ± 5.93
Resp. amplitude (%)	100.0 ± 0.0	112.9 ± 9.6	128.6 ± 11.7^*^	128.6 ± 9.6^*^	128.1 ± 8.6^*^	120.4 ± 8.7^*^^††^

*Intracranial pressure (ICP), renal sympathetic nerve activity (RSNA), systolic arterial blood pressure (ABPs), diastolic arterial blood pressure (ABPd), heart rate (HR), respiratory rate and respiratory amplitude were calculated in mice during 60 s epochs at different ICP levels: 7 mmHg (baseline), 10 mmHg, 20 mmHg, 30 mmHg, 40 mmHg (plateau), and 9 mmHg (post infusion, i.e., 5 min after the end of the infusion). Data are depicted as mean ± sem. Statistical analysis was carried out using ANOVA*.

* *p < 0.05*,

** p < 0.01 vs. baseline and

† *p < 0.05*,

†† *p < 0.01 vs. plateau*.

**Table 2:** Results of heart rate variability analysis during modest ICP increase in animal (*n* = 14).

	**Baseline ICP**	**Plateau ICP**
ICP (mmHg)	7.33 ± 0.90	38.42 ± 2.13^**^
HF (nu)	25.17 ± 5.64	16.46 ± 5.40^*^
LF (nu)	5.54 ± 1.82	9.03 ± 2.58^**^
LF/HF	1.41 ± 0.82	3.02 ± 1.46^**^

This table gives the results of heart rate variability analysis at baseline and during fluid challenge in mice: mean ± sem values of intracranial pressure (ICP), high frequency power (HF), low frequency power (LF), and LF/HF ratio in normalized units. Significance values (ANOVA) are indicated vs. baseline

* *p < 0.05*,

** *p < 0.01*.

In animal, a modest rise in ICP provoked a significant augmentation in RSNA (*cf*. Table 1, Figures 3, 4). The increase in ICP and RSNA were parallel. At the end of infusion, the drop in ICP was associated with a significant reduction in RSNA reflecting partial reversibility of the ICP-mediated sympathetic activation (*cf*. Table 1, Figures 3, 4). During the ICP rise, ABP remained stable and no change in HR was noticed (*cf*. Table 1). Respiratory rate was unchanged but respiratory amplitude augmented significantly during the ICP rise (*cf*. Table 1). Spectral analysis of HR variability (*cf*. Table 2) showed that the rise in ICP was significantly associated with a decrease in HF power (parasympathetic index), whereas LF power and LF/HF ratio increased. This pattern of vagal withdrawal is in accordance with the RSNA results. In mice a modest ICP increase augments *pari passu* efferent SNS outflow, and an ICP drop reduces SNS outflow.

### Effect of modest ICP increase on sympathetic activity in human

**Figure 5:**
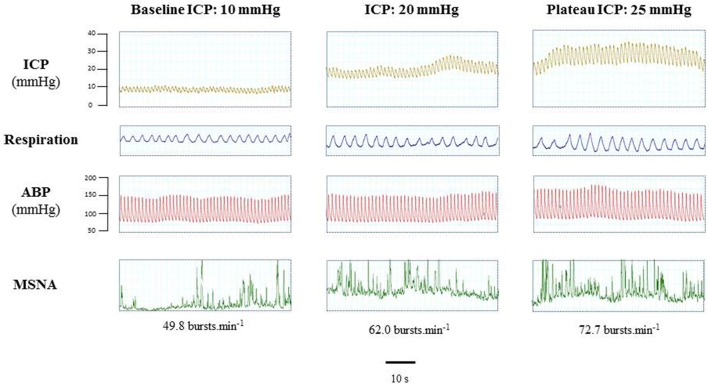
Example of a patient recording with raw signals of ICP, respiration, ABP and sympathetic activity during modest ICP rise. Example of a patient raw signal of ICP, respiration, ABP and neurogram of MSNA at baseline ICP (≈ 10 mmHg), ICP (≈ 20 mmHg) and plateau ICP (≈ 25 mmHg).

**Figure 6:**
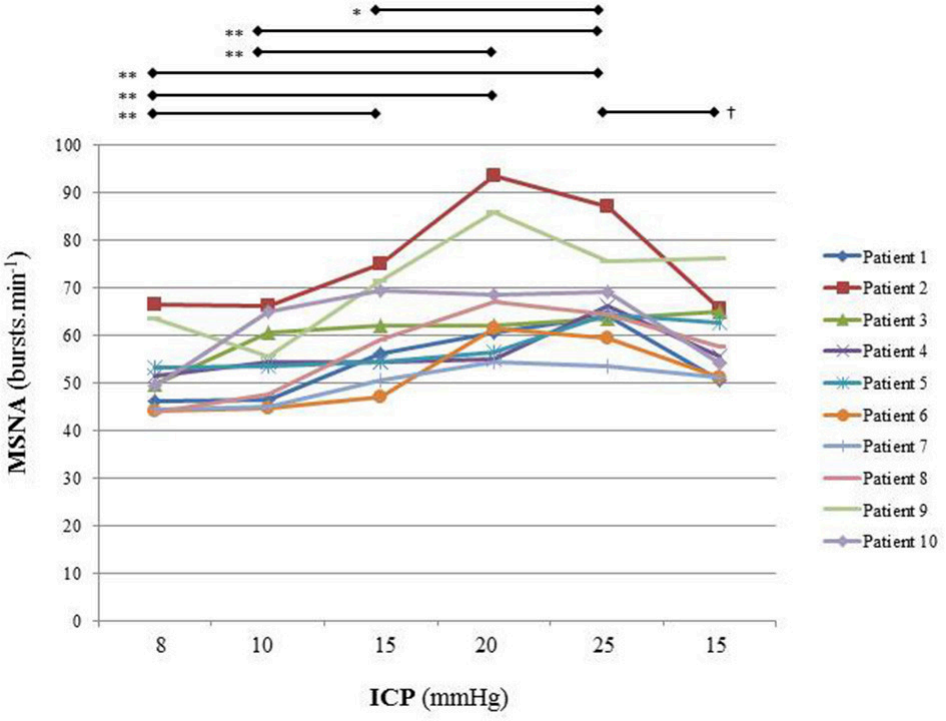
Individual sympathetic activity in each patient during modest ICP increase and decrease (*n* = 10). Statistical analysis was performed using ANOVA for repeated measures with Tukey's post-test. ^*^p < 0.05, ^**^p < 0.01 vs. baseline and ^†^*p* < 0.05 vs. plateau.

**Figure 7:**
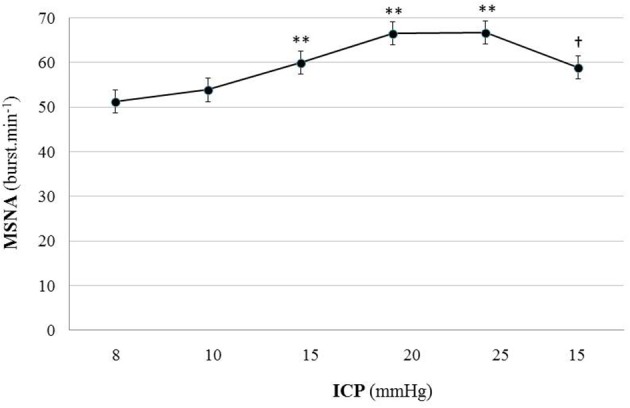
Graph of MSNA changes during modest ICP increase and decrease in human (*n* = 10). Mean ± sem values of MSNA values at various ICP levels. Statistical analysis was performed using ANOVA for repeated measures with Tukey's post-test. ^**^p < 0.01 vs. baseline and ^†^*p* < 0.05 vs. plateau.

Figure 5 is an example of a patient recording with raw signals of ICP, respiration, ABP and MSNA. Figure 6 displays individual MSNA results in each patient during modest ICP increase and decrease. Figure 7 is a graph of MSNA vs. ICP. Table 3 shows human results (MSNA, systolic and diastolic ABP, HR, oxygen saturation, respiratory rate and amplitude) during modest ICP increase and decrease. Table 4 depicts the results of spectral analysis of HR variability at baseline and plateau ICP in patients. Statistical significance is indicated in Figures 6, 7 and Tables 3, 4.

**Table 3:** Results of sympathetic activity and systemic parameters during modest ICP increase and decrease in human (*n* = 10).

**ICP**	**Baseline**	**10 mmHg**	**15 mmHg**	**20 mmHg**	**Plateau**	**Post infusion**
CSF Pressure (mmHg)	8.28 ± 1.05	10.72 ± 0.40	15.64 ± 0.22	20.43 ± 0.31^**^	25.05 ± 0.33^**^	14.83 ± 0.91^†^
MSNA (bursts.min^−1^)	51.24 ± 2.52	53.85 ± 2.54	59.95 ± 2.96^**^	66.50 ± 4.17^**^	66.70 ± 2.88^**^	58.85 ± 2.62^*^^†^
MSNA (bursts /100 HB)	82.86 ± 3.16	88.04 ± 5.54	98.60 ± 5.47^**^	107.50 ± 5.54^**^	106.00 ± 6.57^**^	96.23 ± 4.41^**^^†^
ABPs (mmHg)	145.89 ± 9.56	148.04 ± 9.96	149.64 ± 9.42	150.43 ± 9.97	153.28 ± 10.04	152.67 ± 10.67
ABPd (mmHg)	74.57 ± 3.83	75.36 ± 3.84	74.41 ± 3.84	74.41 ± 3.78	77.10 ± 3.18	77.79 ± 4.08
HR (beats.min^−1^)	62.39 ± 3.54	62.44 ± 3.75	61.63 ± 3.30	62.37 ± 3.52	63.04 ± 3.66	63.44 ± 3.17
Oxygen saturation (%)	94.98 ± 1.03	94.96 ± 0.92	94.99 ± 0.96	94.96 ± 0.87	95.45 ± 0.87	95.85 ± 0.91
Respiratory rate (min^−1^)	17.06 ± 1.02	17.10 ± 1.06	17.26 ± 1.13	17.18 ± 1.07	17.01 ± 1.11	17.36 ± 1.07
Respiratory amplitude (%)	100.0 ± 0.0	103.7 ± 1.4	118.2 ± 11.0	129.6 ± 12.4	133.6 ± 14.0^*^	123.0 ± 12.7

*Cerebrospinal fluid (CSF) pressure, muscle sympathetic nerve activity (MSNA) expressed in bursts.min^-1^ and bursts/100 HB, systolic arterial blood pressure (ABPs), diastolic arterial blood pressure (ABPd), heart rate (HR), oxygen saturation, respiratory rate and respiratory amplitude were calculated in patients during 60 s epochs at different ICP levels: 8 mmHg (baseline), 10 mmHg, 15 mmHg, 20 mmHg, 25 mmHg (plateau), and 15 mmHg (post-infusion, i.e., 5 min after the end of the infusion). Values are depicted as mean ± sem. Statistical analysis was carried out using ANOVA*.

* *p < 0.05*,

** p < 0.01 vs. baseline and

† *p < 0.05*,

†† *p < 0.01 vs. plateau*.

**Table 4:** Results of heart rate variability analysis during modest ICP increase in human (*n* = 10).

	**Baseline ICP**	**Plateau ICP**
CSF Pressure (mmHg)	8.68 ± 0.97	25.41 ± 3.29^**^
HF (nu)	23.59 ± 4.53	17.19 ± 3.81^*^
LF (nu)	33.68 ± 3.29	40.87 ± 4.55^*^
LF/HF	2.01 ± 0.30	3.48 ± 0.79^*^

This table gives the results of HR variability analysis at baseline and during fluid challenge in patients: mean ± sem values of CSF pressure, high frequency power (HF), low frequency power (LF), and LF/HF ratio in normalized units. Significance values (ANOVA) are indicated vs. baseline

* *p < 0.05*,

** *p < 0.01*.

In human, a modest rise in ICP provoked a significant augmentation in MSNA (*cf*. Table 3, Figures 6, 7). The increase in ICP and MSNA were parallel. At the end of infusion, the drop in ICP was associated with a significant reduction in MSNA reflecting partial reversibility of the ICP-mediated sympathetic activation (*cf*. Table 3, Figures 6, 7). During the ICP rise, ABP remained stable and no change in HR or oxygen saturation was noticed (*cf*. Table 3). Respiratory rate was unchanged but respiratory amplitude augmented significantly during the ICP rise (*cf*. Table 3). Spectral analysis of HR variability (*cf*. Table 4) showed that the rise in ICP was significantly associated with a decrease in HF power (parasympathetic index), whereas LF power and LF/HF ratio increased. This pattern of vagal withdrawal is in accordance with the MSNA results. As in mice, in patients a modest ICP increase augments *pari passu* efferent SNS outflow, and an ICP drop reduces SNS outflow.

## Discussion

Using direct gold-standard measurement of sympathetic activity, we demonstrate for the first time in animal and human that ICP is a reversible determinant of efferent sympathetic outflow, even at relatively low ICP levels. Mice and patients results are alike. ICP is a biophysical stress related to the forces within the brain. But ICP has also to be considered as a physiological stressor, driving sympathetic activity. Our data suggest a new neuronal control positively and reversibly linking ICP with sympathetic outflow. Our data point to a possible novel intracranial (i.e., central) baroreflex.

**Figure 8:**
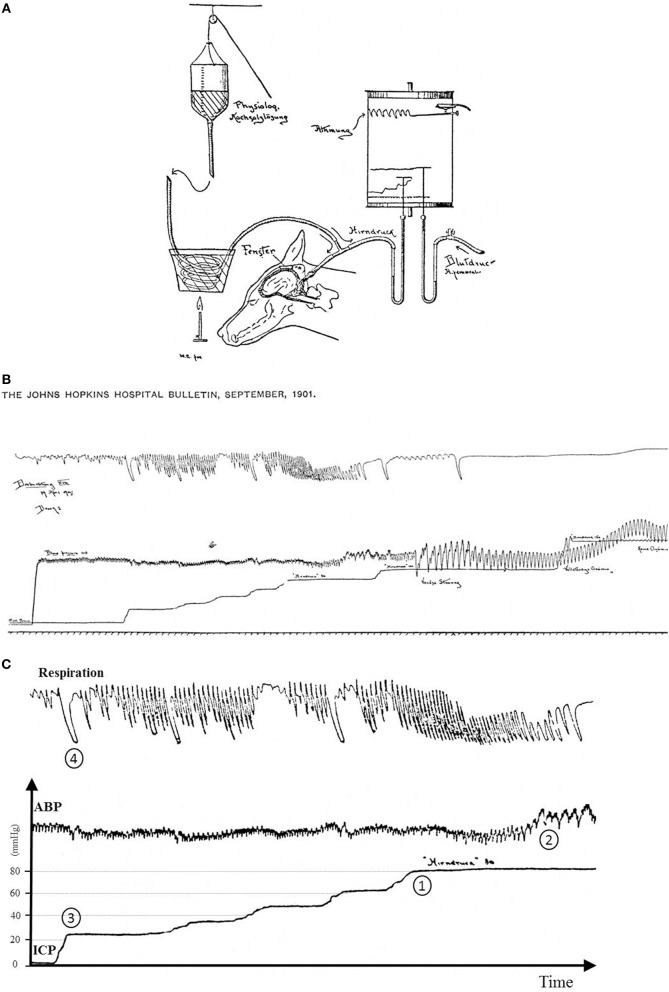
**(A)** Sketch of the original experimental setting from Cushing (1901). A needle was inserted in the *cisterna magna* of a dog to measure the CSF pressure assumed to be the pressure in the brain (*Hirndruck*). The needle connected *via* a tube to a bottle filled with warm saline (*physiologische Kochsalzlösung*). ABP was gauged with an arterial line (*Blutdruck Arteria femoralis*). Fluid pressures were measured with mercury tubes. Respiration was also measured (*Atmung*). All signals were recorded on a rotating paper system. ICP was stepwise increased by lifting the bottle with a rope. **(B)** Original recording from Cushing (1901). From the bottom to the top are displayed respectively traces of respiration, ICP and ABP. ICP was stepwise increased. It was possible to retrieve pressure values since *Hirndruck 80* and *Hinrdruck 100* were handwritten on the chart. To facilitate the interpretation of the recording, we reframed, annotated and added scales in **(C)**. **(C)** Original recording from Cushing (1901) with annotations and scales. The Cushing response was elicited once ICP reached 80 mmHg (marker ①) with a rise in ABP (marker ②). Note the latency between ICP rise and the ABP response. But when ICP was increased from baseline to ≈20 mmHg (first bump in the ICP recording, marker ③), it yielded a slight change in ABP with an increase in respiration amplitude (marker ④). Note the synchronicity between ICP rise, ABP change and respiratory response. Hence the original recording displays two events: a late event (Cushing response), but also an early one different from the Cushing response.

**Figure 9:**
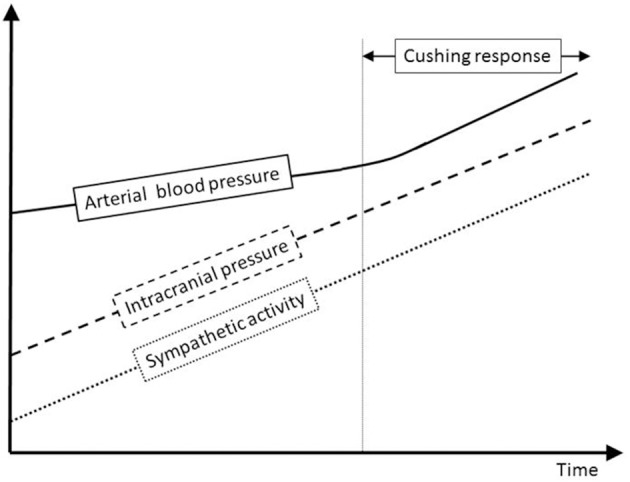
Sympathetic and ABP response to ICP increase. This schematic figure is an attempt to display our hypothesis about the interplay between ICP, sympathetic activity and ABP. Modest ICP increase augments *pari passu* sympathetic activity that parallels the ICP rise, but has a mild influence on ABP. For higher ICP values, the increase in sympathetic activity yields an ABP rise that heralds the Cushing response, very last hemodynamic consequence of a continuous ICP-mediated sympathetic activation.

The Cushing response is an immensely powerful but agonal, terminal and irreversible event due to ischemia of the brain-stem. It produces massive intrinsic (i.e., non-synaptic) activation of the sympatho-excitatory neurons of the ventrolateral medulla (Nagao et al., 1984). In that respect it is not regarded as a reflex but as a “last ditch” protection for an ischemic brain, analogous to the auto-resuscitative mechanism of gasping. We reproduce Harvey Cushing's original experimental recording published in 1901. We display a sketch of the experimental setting in anesthetized dog (*cf*. Figure 8A) and an original recording (*cf*. Figures 8B,C). ICP augmentation close to ABP (*cf*. marker ① in Figure 8C) results in an ABP rise and respiratory irregularities i.e., a Cushing response (*cf*. marker ② in Figure 8C). Note the time delay between the ICP rise (marker ①) and the ABP response (marker ②). However, close analysis of the initial part of this recording shows that a modest ICP increase from 0 to ≈20 mmHg (*cf*. marker ③ in Figure 8C) yields immediate fluctuation of ABP and deep breath with increase in respiratory amplitude (*cf*. marker ④ in Figure 8C). Note the synchronicity between ICP rise (marker ③), ABP fluctuation and respiratory change (marker ④). This is in accordance with our experimental results in mice and patients. More than a century ago, Harvey Cushing was evidently recording sympathetic activation during modest ICP rise in dog. It seems that the Cushing response is the tip of an iceberg, very last hemodynamic consequence of a continuous sympathetic activation that parallels the ICP rise. Historical and modern literature suggest the presence of an intracranial (i.e., central) baro-sensitive mechanism regulating efferent sympathetic activity (Dickinson, 1990; Paton et al., 2009; McBryde et al., 2017), but direct evidence was still lacking. Our data seem to show that sympathetic activity parallels ICP changes. We propose to summarize our hypothesis with a simple graph (*cf*. Figure 9). From our data we propose a unified physiological concept that might correspond to an intracranial (i.e., central) baroreflex, with possible implication in the regulation of sympathetic activity set-point. Indeed SNS plays a significant role in ABP short-term regulation, mainly by extracranial (i.e., peripheral) baroreflex mechanisms (Cowley et al., 1973) but also by complex homeostatic feedback mechanisms (Dampney et al., 2002). In contrast to the sympatho-inhibitor extracranial baroreflex, this novel intracranial baroreflex seems to be sympatho-activator. Hence, intracranial and extracranial baroreflex might act in opposition on the SNS. The former activates but the latter inhibits the sympathetic activity, as a fine-tuning regulatory mechanism of SNS. This represents a paradigm shift in the physiology of heart/brain regulatory cross-talk with novel insight into the pathophysiology of cardio-metabolic homeostasis imbalance. The paradoxical decrease in cardiac baroreflex sensitivity due to vagal modulation in the upright posture (Silvani et al., 2017) or the alteration of cerebral autoregulation in subjects prone to syncope (Bari et al., 2017) might be in line with our hypothesis.

We demonstrate in mice and patients that during ICP augmentation, both RSNA and MSNA increase prior to any significant ABP rise. In a previous study we analyzed changes in ABP during ICP rise in a larger group of similar patients (*n* = 34) but without MSNA measurement (Schmidt et al., 2005). In this latter study ICP increase (from 8.2 ± 0.9 to 25.0 ± 1.4 mmHg), had a statistically significant effect on both systolic ABP (145.3 ± 6.2 to 156.7 ± 6.4 mmHg) and diastolic ABP (76.5 ± 5.4 to 90.5 ± 4.5 mmHg). In our current group, we only identified a trend in ABP increase (*cf*. Table 3). In the present study, the reduced sample size (*n* = 10) probably explains the lack of power and the absence of statistical significance. We know from normotensive subjects with high levels of baseline MSNA that a relatively low value of cardiac output can offset the influence of higher sympathetic outflow and vascular resistance (Charkoudian et al., 2006). Therefore, the increase in MSNA or RSNA without substantial ABP rise suggests either a parallel opposing vasodilator mechanism or latency between SNS augmentation and systemic response.

ICP-mediated sympathetic activation may result either from a baro-sensitive or a chemo-sensitive mechanism. The baro-sensitive mechanism is supported by the presence of pressure-sensitive areas in the brainstem with ability to trigger a sympathetically-mediated systemic response (McBryde et al., 2017). In anesthetized dogs, direct solid stress on the surface of the brainstem without altering ICP produced an increase in ABP (Thompson and Malina, 1959). Pressure receptive area was more precisely identified in the lower brainstem (Hoff and Reis, 1970; Doba and Reis, 1972; Reis and Doba, 1972). A subpial catecholaminergic neuronal group was identified in the rostroventral medulla yielding a pressor response when stimulated mechanically (Dampney et al., 1982; Jannetta et al., 1985). In human autopsy series, a similar population of catecholaminergic neurons has been identified in the subpial regions of the retro-olivary sulcus (Aicher et al., 1996). The baro-sensitive mechanism hypothesis is also in line with the concept of “neurogenic” origin of hypertension (Jannetta et al., 1985) since mechanical compression of the lateral part of the medulla can play a role in ABP elevation (Reis and Doba, 1972). The ICP-mediated systemic response seems to be rapid, beginning within 1 s of the onset of direct intracranial compression (Rodbard and Stone, 1955). The absence of latency between intracranial stress and sympathetic response also supports the hypothesis of an intracranial baro-sensitivity, since ischaemia/anoxia would take longer to develop (*cf*. marker ③ and ④ in Figure 8C). The chemo-sensitive hypothesis stipulates that hypoxia increases sympathetic tone. The key organs for monitoring the delivery of oxygen to the brain are the extra cranial carotid bodies that are not directly influenced by ICP. The sympathetic adjustments triggered by acute mild hypoxia are initiated by activation of peripheral chemoreceptors whereas more severe hypoxia activates the sympathetic outflow *via* direct effects on the brainstem (Bruce and Cherniack, 1987; Guyenet, 2000). The rapid and reversible ICP-mediated sympathetic activation in awake patients probably points to a mechanic (baro-sensitive) rather than hypoxic (chemo-sensitive) central mechanism. However, the exact mechanism by which ICP modulates sympathetic circuit within the brain stem requires further studies.

We show in mice and patients a parallel increase in ICP, sympathetic activity and respiration amplitude. An ICP drop was also associated with a parallel decrease in sympathetic activity and respiration amplitude (*cf*. Tables 1, 3). From the Cushing experiment, a modest and rapid increase in ICP yielded a simultaneous increase in breath depth (*cf*. marker ④ in Figure 8C). This is in accordance with our animal and human data. Breathing is a motor behavior, generated and controlled by the central nervous system. Breathing fluctuates with sympathetic activity (Dempsey et al., 2002; Jänig and Häbler, 2003). Neurons that generate breathing are arranged in the lateral pons (Alheid et al., 2004) and extend to the rostral ventrolateral medulla (Dutschmann and Dick, 2012). Baro-sensitive neurons that regulate sympathetic efference also extend to the rostral ventrolateral medulla and are strongly inhibited by activation of peripheral (i.e., extracranial) arterial baroreceptors and higher centers in the brain (Dampney et al., 2002; Guyenet, 2006). Hence the rostral ventrolateral medulla seems to be a nodal point for ABP and respiration regulation. Previous work already suggested that peripheral chemoreflex and baroreflex share common interconnecting pathways (Despas et al., 2012). Our data support the concept of a central cardiorespiratory integration network.

Sympathetic activity is responsible for priming the body for action, particularly in stress situations threatening survival. Sympathetic over-activity is associated with the initiation, progression and prognosis of various disorders like arterial hypertension, heart and renal failure, diabetes mellitus or obesity (Grassi et al., 2007). In patients, we show that a modest 7 mmHg ICP rise (from 8 to 15 mmHg) significantly increases sympathetic activity by 17% (from 51.2 to 60 bursts.min^−1^) without ABP change (*cf*. Table 3). A 7 mmHg ICP increase occurs very frequently in everyday life of healthy individuals, for example when lying down from the upright to the horizontal position (Magnaes, 1976). Our hypothesis is also supported by the demonstration that acute head down tilt test is associated with an enlargement of the optic nerve sheath diameter reflecting ICP rise, and an increase in sympathetic activity using MSNA recording (Kermorgan et al., under review). Similar or higher ICP increases are encountered in various pathological conditions such as head trauma, hydrocephalus, stroke and airway obstruction with sleep apnoea (Rangel-Castilla et al., 2008). After a stroke, sympathetic over-activity is reported with increased risk of cardiovascular complications and poor outcome (Hilz et al., 2011). Takotsubo cardiomyopathy, an acute heart failure attributed to a surge in catecholamine levels, is reported after intracranial hemorrhage with intracranial hypertension (Abd et al., 2014). Obesity and weight gain, that represent major risk factors for obstructive sleep apnoea, are also associated with ABP and MSNA increases (Narkiewicz et al., 1998). In those acute or chronic conditions, the exact role of modest ICP rise on sympathetic overdrive has to be addressed.

There are several limitations to our study. First, the size of the study populations is rather small. However, we used RSNA and MSNA as the main criteria to assess SNS activity and these parameters have a reduced intra-individual variability making them suitable for such limited population size. Second, included patients were not normal subjects but patients suspected of hydrocephalus. Even though confirmation in normal volunteers is necessary to demonstrate that an intracranial baroreflex is part of normal physiology, for ethical reasons it was more appropriate at this stage to carry out this first study in patients. Third, although our data demonstrate that ICP regulates efferent SNS drive, additional work is necessary to identify the afferent SNS activation pathways and the intracranial baroreceptor underlying SNS modulation. Finally, it is necessary to investigate the time-course and persistence of this novel highly-regulated SNS modulatory circuit, as well as its relevance in terms of clinical practice.

In line with the composite nature of baroreflex (Marchi et al., 2016b; Reyes Del Paso et al., 2017) our data pave the way for further work in order to (i) investigate ICP as a new arm of baroreflex, (ii) validate the hypothesis of an intracranial baroreflex, (iii) identify the afferent pathway and the cardiorespiratory integration centers, (iv) demonstrate that ICP modulates sympathetic activity set-point, (v) investigate the role of raised ICP in the pathogenesis of sympathetic over-activity, (vi) explore the possible sympatholytic effect of reducing ICP, and finally (vii) determine whether ICP control, e.g., with a drug or a ventricular shunt, should be clinically relevant in sympathetically-driven diseases.

## Ethics statement

The animal study was approved by the University of Iowa Animal Research Committee. The human study was approved by the competent authority (N° ANSM 2007-A01430-53), by local Ethics committee (N° 2008-0044) and registered on ClinicalTrials.gov under n° NCT01776801. Patients gave their written consent for participation to the study.

## Author contributions

The animal experiments have been done in the Departments of Pharmacology, University of Iowa, Iowa City, Iowa, USA. The human study has been performed in the Autonomic Unit of the University Hospital of Toulouse, Toulouse, France. ES was PI of the study. He conceived and designed the project, included the patients in the study, acquired mouse and human data. He was the main writer of the paper. FD was involved in acquisition and analysis of the microneurography in mice and human. He did the statistical analysis and revised the paper. AP-LT was responsible for acquisition and analysis of the heart rate variability. She also revised the paper. ZC and JP were important intellectual collaborators. They conceived the concept of the work and thoroughly revised the paper. KR supervised the animal work and revised the paper. AP was involved in the acquisition of microneurography in human, and designed the animal study. He revised the paper. JS was responsible for the draft, critical approach and intellectual content of the work. He revised the manuscript in details. All authors approved the final version of the manuscript and agree to be accountable for the accuracy or integrity of any part of the work. All persons designated as authors qualify for authorship, and all those who qualify for authorship are listed.

### Conflict of interest statement

The authors declare that the research was conducted in the absence of any commercial or financial relationships that could be construed as a potential conflict of interest.

## Abbreviations

ABP: arterial blood pressure
CPP: cerebral perfusion pressure
CSF: cerebrospinal fluid
FFT: fast Fourier transform
HB: heart beats
HF: high frequency of heart rate variability analysis
HR: heart rate
ICP: intracranial pressure
LF: low frequency of heart rate variability analysis
MSNA: muscle sympathetic nerve activity
PI: pulse interval
RSNA: renal sympathetic nerve activity
SNS: sympathetic nervous system
TP: total power of heart rate variability analysis.
